# Inflammatory markers as predictors of mortality in COVID-19 infection

**DOI:** 10.4102/ajlm.v9i1.1298

**Published:** 2020-12-21

**Authors:** Awadia A. Ahmeidi, Ashraf Musa, Hend S. Ahmed, Adel A. Elahmar, Ryhana B. Goota, Ibtihal A. Ahmed, Abdelhakam H. Ali, Mushal Allam, Mozan O. Hassan

**Affiliations:** 1 Department Hematology, Faculty of Medical Laboratory Science, University of Science and Technology, Khartoum, Sudan; 2 Abeer Hospital, Muscat, Oman; 3 Department Hematology and Blood Transfusion, Faculty of Medical Laboratory Science, Omdurman Ahlia University, Khartoum, Sudan; 4 Communicable Disease Center, Harmad Medical Corporation, Doha, Qatar; 5 Ministry of Health, Khartoum, Sudan; 6 Faculty of Medical Laboratory Science, Ibn Sina University, Khartoum, Sudan; 7 Department Microbiology, Faculty of Medical Laboratory Science, University of Al Butana, Rufaa, Sudan; 8 National Institute for Communicable Diseases, National Health Laboratory Service, Johannesburg, South Africa

Since the origination of the severe acute respiratory syndrome coronavirus-2 (SARS-CoV-2) in China in December 2019 and its subsequent global spread, it became critical to understand the inflammatory process associated with this virus and the coronavirus disease 2019 (COVID-19) and to understand the changes associated with the different inflammatory parameters. The clinical presentation of COVID-19 cases varies greatly from mild symptoms among the majority of patients, to severe disease leading to death in some patients.

Many COVID-19 cases had increased levels of inflammatory cytokines and other infection-related biomarkers.^1^ The inflammatory markers, including interleukin-6, D-dimer, neutrophil-to-lymphocyte ratio and high-sensitivity C-reactive protein (hs-CRP) levels, were found to be indicative of severe COVID-19 in reports that emerged from China.^2^ However, their association with mortality among COVID-19 patients has not been reviewed. In this article, we identify inflammatory markers (interleukin-6, D-dimer, neutrophil-to-lymphocyte ratio and hs-CRP) as predictors of COVID-19 mortality. Laboratory tests for these markers are simple, cheap and available and can be performed in outbreak areas with limited resources.

According to Zhou et al., D-dimer levels exceeding 1.0 *µ*g/mL at hospital admission correlated significantly with death among hospitalised COVID-19 patients in China, with a *p*-value of less than 0.001.^3^ Another study in Wuhan, China, which included 343 in-patients with confirmed COVID-19, showed that elevated D-dimer levels over 2 *µ*g/mL at an early stage of hospitalisation correlated significantly with a high risk of death.^4^ Also, according to another study from China that included 1099 confirmed COVID-19 patients, results revealed that median D-dimer and C-reactive protein levels were higher among severe cases compared to non-severe cases, demonstrating that high D-dimer and C-reactive protein levels were significantly associated with COVID-19 severity.^5^ Additionally, in Wuhan Jinyintan Hospital, China, a study showed that high levels of interleukin-6 were significantly associated with a high mortality rate.^6^ This finding was corroborated by Silberstein in this study.^7^ Liu Y et al. in Zhongnan Hospital of Wuhan University identified an elevation in the neutrophil-to-lymphocyte ratio as an independent and significant predictor of mortality among 245 hospitalised COVID-19 patients, with an 8% increase in mortality with each unit increase in neutrophil-to-lymphocyte ratio.^8^

Many studies reported a correlation between high levels of hs-CRP and mortality rate in COVID-19 patients. A study done among 375 patients with confirmed SARS-CoV-2 infection revealed that elevated hs-CRP levels were significantly associated with a high mortality risk.^9^ In another study conducted to evaluate fatal outcomes among COVID-19 patients, 187 patients from China were included among whom 43 died, and results revealed that high levels of hs-CRP was significantly associated with mortality.^10^

We conclude that elevation in levels of these four inflammatory markers may be indicative of COVID-19 infection severity and mortality. We suggest that these parameters may be helpful predictors of COVID-19 severity and could be used as early predictors for case management before deterioration. On the other hand, these parameters cannot be used independently for initial diagnosis and physicians need to monitor the presence of other infections that may interfere with the elevation of these markers. Finally, we recommend that similar studies on inflammatory markers should be conducted in African populations to demonstrate the levels of these markers among African COVID-19 patients and their association with COVID-19 severity and mortality.
